# A novel method for processing adipose-derived stromal stem cells using a closed cell washing concentration device with a hollow fiber membrane module

**DOI:** 10.1007/s10544-020-00541-0

**Published:** 2021-01-06

**Authors:** Shinji Hayashi, Rieko Yagi, Shuhei Taniguchi, Masami Uji, Hidaka Urano, Shinya Yoshida, Hiroshi Sakurai

**Affiliations:** 1 Biomaster, Inc., Yokohama, Japan; 2 Medical Solutions Vehicle, Kaneka Corporation, 5-1-1, Torikai-nishi, Settsu, Osaka, 566-0072 Japan

**Keywords:** Adipose-derived stromal stem cells, Stromal vascular fraction, Cell-assisted lipotransfer, Closed cell washing concentration device, Hollow fiber membrane module

## Abstract

Cell-assisted lipotransfer (CAL) is an advanced lipoinjection method that uses autologous lipotransfer with addition of a stromal vascular fraction (SVF) containing adipose-derived stromal stem cells (ASCs). The CAL procedure of manual isolation of cells from fat requires cell processing to be performed in clean environment. To isolate cells from fat without the need for a cell processing center, such as in a procedure in an operation theater, we developed a novel method for processing SVF using a closed cell washing concentration device (CCD) with a hollow fiber membrane module. The CCD consists of a sterilized closed circuit, bags and hollow fiber, semi-automatic device and the device allows removal of >99.97% of collagenase from SVF while maintaining sterility. The number of nucleated cells, ASCs and viability in SVF processed by this method were equivalent to those in SVF processed using conventional manual isolation. Our results suggest that the CCD system is as reliable as manual isolation and may also be useful for CAL. This approach will help in the development of regenerative medicine at clinics without a cell processing center.

**Supplementary Information:**

The online version contains supplementary material available at 10.1007/s10544-020-00541-0.

## Introduction

Mastectomy and breast-conserving surgery in treatment of breast cancer are accompanied by changes in the shape of the breast, leading to poor quality of life (QOL) due to mental distress and inconvenience in daily life. To recover QOL for patients after breast cancer treatment, breast reconstruction can be performed using plastic surgery. In Japan, the annual rates of simple mastectomy increased from 5.6% in 2004 to 13.0% in 2011, and this has been attributed to an increased rate of breast reconstruction in recent years (Kurebayashi et al. 2015). Breast reconstruction with autologous adipose tissue has advantages of natural softness and body temperature, no large scars in the collection and transplant areas, an improved shape of the indentations under the clavicle, and ease of formation. However, because of ischemia, all of the grafted fat does not survive, and cysts or fibrosis can develop (Mineda et al. 2014).

Cell-assisted lipotransfer (CAL) (Matsumoto et al. 2006; Yoshimura et al. 2008) is an advanced lipotransfer method for breast reconstruction. In CAL, grafting fat is enriched with adipose-derived stromal stem cells (ASCs) contained in the stromal vascular fraction (SVF) obtained after enzymatic digestion of autologous adipose tissue. Zuk et al. (2002) showed that ASCs have the capacity to differentiate into various cell lineages. In turnover of adipose tissue, ASCs act as progenitors of adipocytes and vascular cells (Planat-Benard et al. 2004) and release angiogenic growth factors such as HGF and SDF-1 in response to injury, hypoxia and other conditions (Rehman et al. 2004; Thangarajah et al. 2009; Suga et al. 2009). These effects of ASCs are believed to improve survival of transplanted fat, and the safety and efficacy of CAL have been shown in several studies (Yoshimura et al. 2010; Pérez-Cano et al. 2012; Laloze et al. 2017). In addition to breast reconstruction, SVF has been used clinically for a variety of indications, including osteoarthritis, sclerosis, tendinopathy, congestive heart failure, chronic obstructive pulmonary disease, and radiation necrosis (Comella et al. 2017).

Isolation of SVF for clinical use from adipose tissue requires a tightly controlled cell processing center (CPC) and trained technicians to ensure sterility, safety, quality, and stability. An automated SVF isolation system is required to reduce human error, labor, and building costs, and to maintain the CPC and facilitate clinical trials. The Celution® system (Cytori Therapeutics, CA, USA) and Icellator® (Tissue Genesis, HI, USA) are automated devices for SVF isolation from adipose tissue, which use conventional cell isolation by enzymatic digestion and centrifugal cell washing. Centrifugation is a general method for cell washing or concentration, but repeated centrifugation may damage cells. We focused on development of a closed cell washing and concentration device (CCD) with a hollow fiber membrane module that does not require centrifugation and opening operations. The CCD uses a tangential flow filtration, a method in which a solution is flowed in parallel on the membrane and pressure is applied perpendicularly to the membrane to apply filtration and to wash and concentrate cell suspensions to maintain cell dispersibility. A CCD is likely to cause less cell damage due to the lack of centrifugal force and to reduce the risk of contamination due to fewer opening operations.

In this study, we evaluated the efficiency and reliability of SVF processing using the CCD. The safety of the CCD system was evaluated by measuring the amount of residual enzyme and general bacteria in the isolated SVF. SVFs obtained using conventional manual centrifugation and the CCD system were compared based on the number of nucleated cells, viability, and ASC contents.

## Ethical considerations

This study was approved by ethical association of Biomaster. Moreover, CAL treatment has been accepted by the Ministry of Health, Labour and Welfare, Japan. Informed consent was obtained from each patient.

## Materials and methods

### Adipose tissue collection

Human aspiration fat was obtained from patients (*n* = 14, 1 male and 13 females) during plastic surgery at the Cellport Clinic Yokohama (Kanagawa, Japan). Lipoaspirate was harvested from a pre-specified site (abdomen, thigh, etc.) using a 3–4 mm blunt tip cannula and centrifuged at 700×*g* for 5 min, after which excess blood were removed. Lipoaspirates from four donors were equally divided and used for comparison of the CCD system and the manual centrifugation method.

### Lipoaspirate processing

**Fig. 1:**
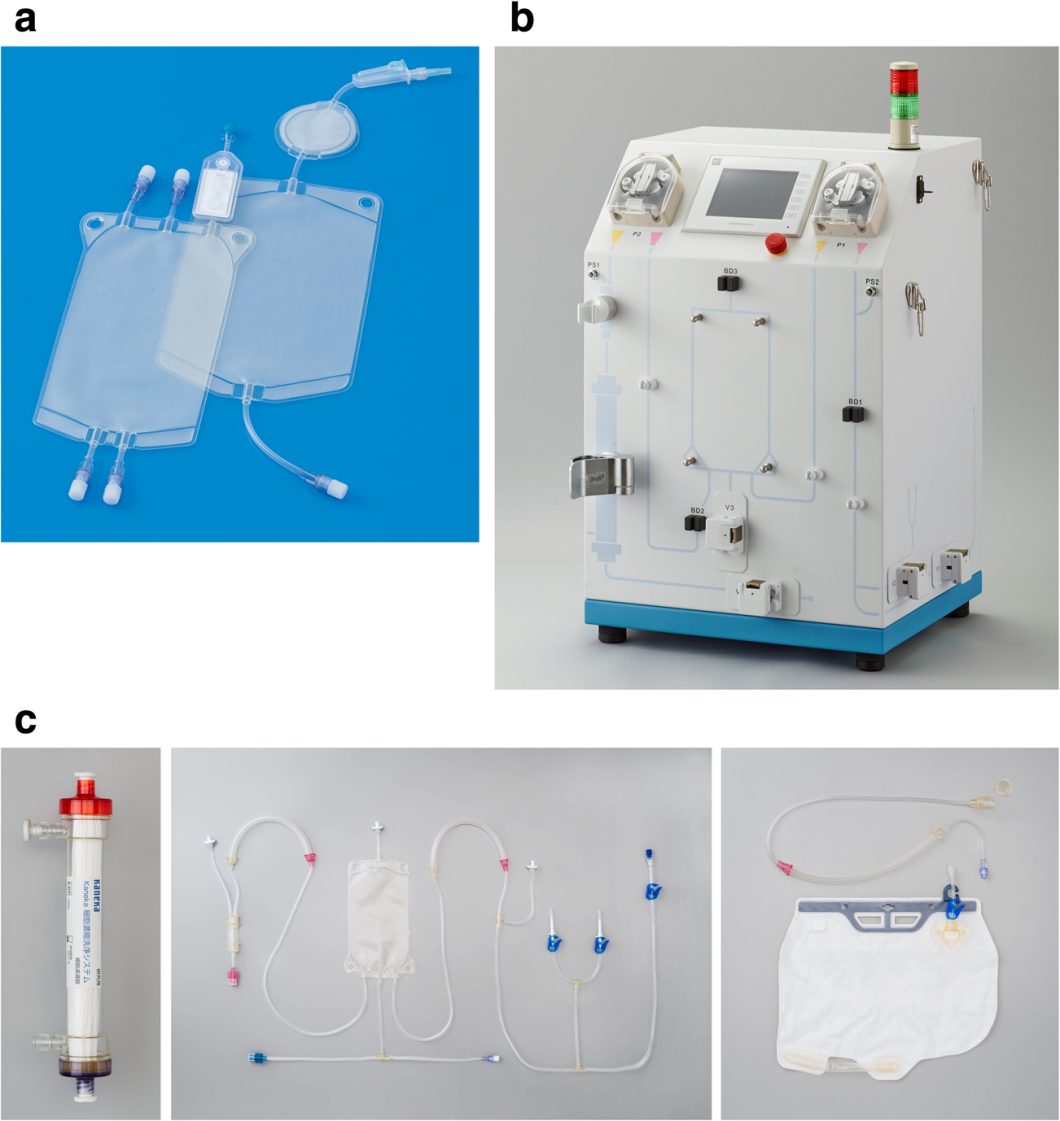
The CCD system. (**a**) Single-use disposable lipoaspirate processing kit (left: Bag A for lipoaspirate digestion, right: Bag B for aqueous layer collection). (**b**) Cell washing concentration processing device. (**c**) Single-use disposable washing concentration circuit kit (left hollow fiber membrane module, center: tubing circuit for circulation side, right: tubing circuit for filtrate side)

**Fig. 2:**
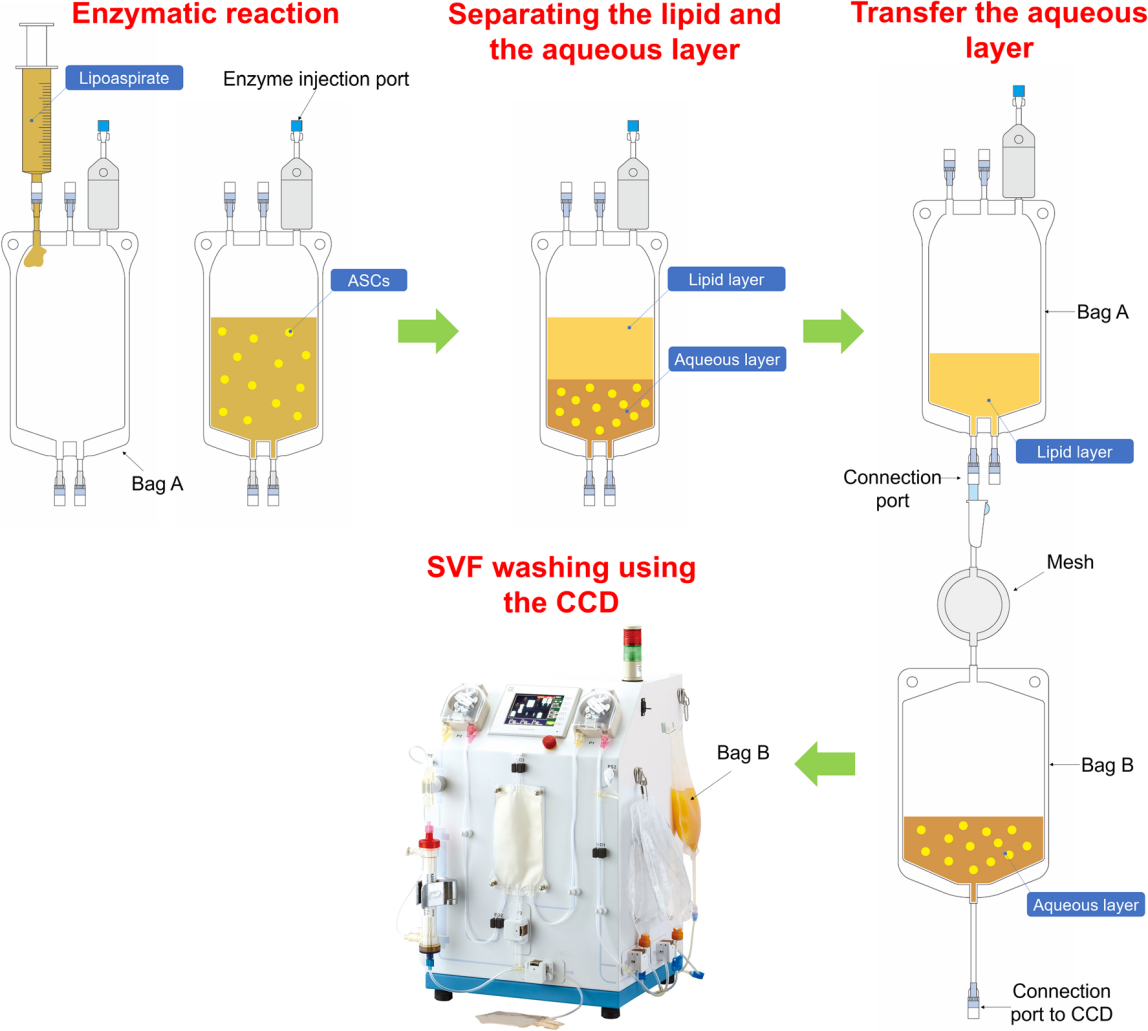
Schematic representation of the lipoaspirate processing using the CCD system

SVF processing using the CCD consists of lipoaspirate digestion and cell washing concentration using a closed cell washing concentration system (Kaneka, Japan) (Fig. 1). The kit for lipoaspirate processing and the washing concentration circuit kit are single-use disposable. SVF was processed according to the instruction manual in each kit. Schematic representation of the lipoaspirate processing is shown in Fig. 2. In brief, lipoaspirate (20–200 mL) was injected into Bag A and digested in the same volume of HBSS containing 0.2% collagenase (Worthington Biochemical, USA) on a shaker at 37 °C for 30 min. After digestion, Bag A was kept upright for 5 min to separate into a lipid layer containing mature adipocytes and oil, and an aqueous layer containing ASCs. Bag A was connected to Bag B, and the lower aqueous layer from Bag A was transferred to Bag B through the filter by gravity. The lipid layer in Bag A was washed with Lactated Ringer’s solution, and the washed aqueous layer was also collected in Bag B. Washing was repeated 3 times. Bag B was connected to the CCD, and SVF was washed and concentrated automatically. The final product was approximately 20 ml of SVF (CCD-SVF). The whole process time using the CCD system was about 120 min.

For comparison with the CCD system, SVF was manually isolated from lipoaspirate (Yoshimura et al. 2006). Briefly, lipoaspirate was digested with the same volume of HBSS containing 0.2% collagenase (the same product used for the CCD system above) on a shaker at 37 °C for 30 min. After digestion, mature adipocytes and oil were separated from pellets by centrifugation (800×*g* for 10 min). The pellets were resuspended with Lactated Ringer’s solution and centrifuged (800×*g* for 5 min) three times to obtain SVF (manual-SVF). The manual SVF processing method took about 100 min.

### Collagenase removal analysis

For evaluation of collagenase removal by CCD, the amounts of collagenase in SVF before and after CCD processing were measured using an EnzChek gelatinase/collagenase assay kit (Invitrogen Life Tech), and the removal rate was calculated as follows. Collagenase removal rate (%) = (A - B) / A × 100, where A and B are the amounts of collagenase in SVF before and after CCD processing, respectively. Measurements were performed three times for each sample and the average of the triplicate data is shown as the sample data.

### Isolated cell analysis

To determine the cell composition of SVF, we used multicolor flow cytometric analysis to examine surface marker expression on the cells in SVF. The following monoclonal antibodies conjugated to fluorochromes were used: anti-CD31-Alexa647, anti-CD34-PE, and anti-CD45-FITC (BD Biosciences, USA). Cells were analyzed with a FACS Canto™ Flow Cytometer (BD Biosciences, USA). CD31-/CD34+/CD45- cells were regarded as ASCs, whereas CD45-/CD31+/CD34+ cells were regarded as endothelial cells (ECs). CD45+ cells were regarded as blood-derived cells (BCs) (Suga et al. 2008). The number and relative viability of nucleated cells in SVF were determined using an automated Cell Counter (NucleoCounter NC-100, Chemometec, Denmark). Counting was performed three times for each sample and the average of the triplicate data is shown for the sample. Three CCD-SVFs were tested commercially (SRL Co., Japan) for sterility according to the Japanese Pharmacopoeia.

### Comparison with clinical results

For comparison with clinical results, the cell yield and viability of SVF from 44 patients in CAL treatment at Cellport Clinic Yokohama from January 2018 to December 2018 were compared to those for CCD-SVF. These patients gave permission for disclosure of these data.

## Results and discussion

The efficiency and reliability of the CCD for SVF isolation from adipose tissue were examined. The CCD was able to remove >99.97% of collagenase in SVF (Table 1). In clinical use of SVF, removal of additional non-cell components, especially added proteins such as collagenase, is very important for the safety of patients. The toxicity of residual collagenase in SVF is uncertain, but in a 4-week toxicity study in NOD/SCID mice using ADSCs obtained by digesting fat with collagenase, residual collagenase depending on the frequency of washing did not show any specific toxicity (Chang et al. 2013). In a suspension of cultivated cell lines containing serum and purified proteins (IgG, IL-2, and myoglobin), we showed that 99% of the protein components can be removed using the CCD (in-house data). The results of this study show that the CCD is effective for washing of non-cultured tissues derived from primary cells.

**Table 1:** The collagenase removal rate processed by CCD system

Donor ID	The collagenase removal rate (%)
1	99.98
2	99.97
3	99.97

There have been no reports on the use of a hollow fiber membrane for tissue-derived cell processing, but blood cell washing and concentration devices have been described (Arnaud et al. 2003; Lu et al. 2019). Cell separation and volume reduction techniques using centrifugation have been shown to damage blood cells and contribute to adverse outcomes. Therefore, membrane filtration technologies, such as a hollow fiber membrane, are attractive alternatives to centrifugation-based cell processing of blood products. Arnaud et al. (2003) successfully removed 95% of DMSO from cryopreserved platelets using a hollow fiber device (PSN 120, Baxter, USA), showing a level of performance similar to that of conventional centrifugation-based washing.

A characteristic of the CCD is cell washing concentration by tangential flow filtration. This method reduces cell aggregation because no centrifugal force is applied. As shown in the Supplemental Figure, we confirmed that the final product after CCD processing has excellent cell dispersibility (in-house data). This cell dispersibility using the CCD is thought to contribute to the washing efficiency.

**Fig. 3:**
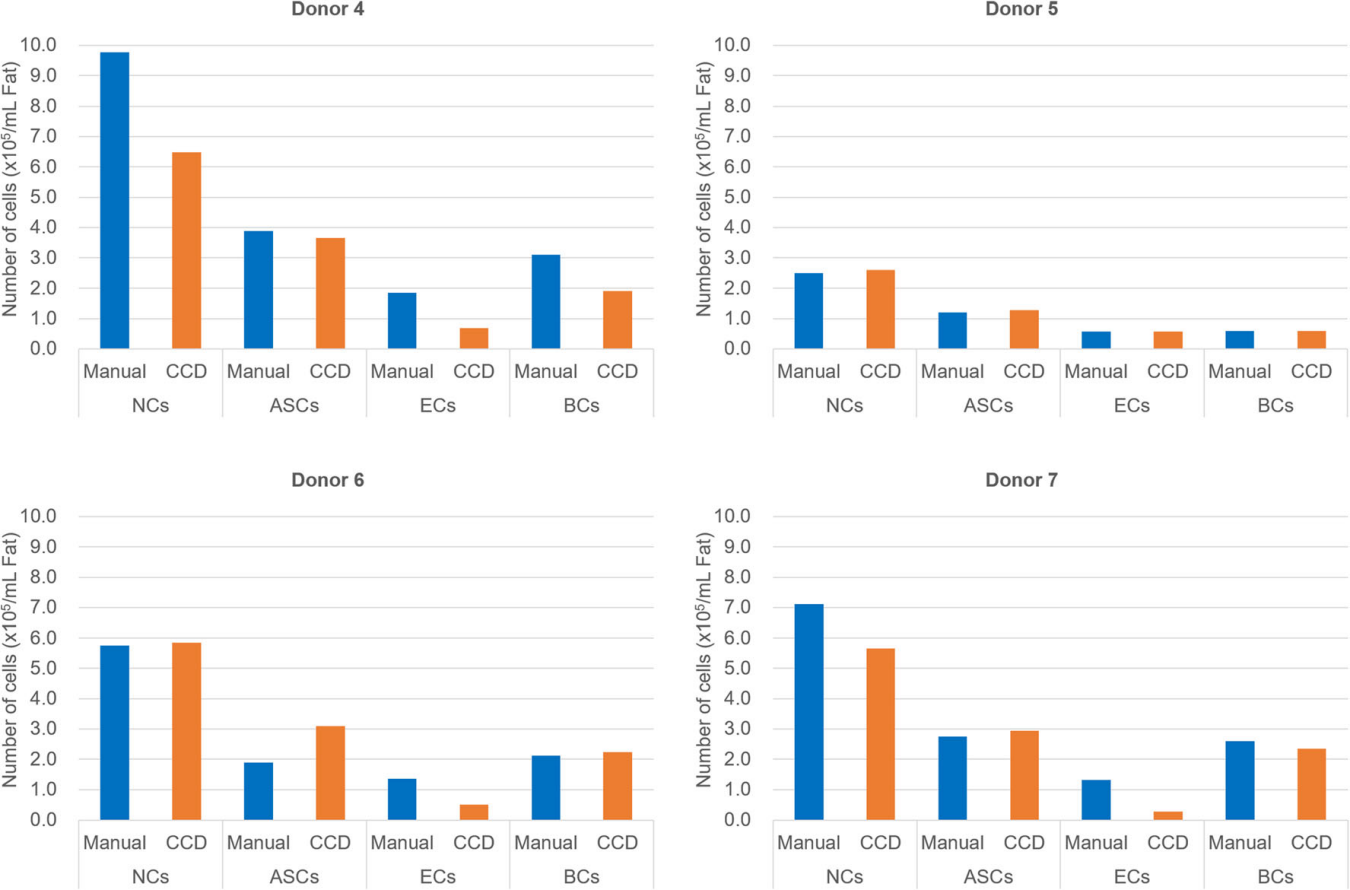
Flow cytometry analysis of SVF processed by conventional manual centrifugation (Manual) and the CCD system (CCD). Abbreviations: NCs, nucleated cells. ASCs, adipose-derived stromal stem cells (CD31-/CD34+/CD45- cells). ECs, endothelial cells (CD31+/CD34+/CD45- cells). BCs, blood Cells (CD45+ cells)

Manual-SVF and CCD-SVF isolated from adipose tissue from the same patient were compared in terms of cell yield and composition. In manual-SVF and CCD-SVF, the numbers of nucleated cells were 6.02 ± 3.02 × 10^5^ and 5.15 ± 1.73 × 10^5^ cells/mL fat, respectively (*n* = 4), and the viabilities were 85.15 ± 3.35% and 75.25 ± 2.40%, respectively. There were more ASCs in CCD-SVF (52.58 ± 3.06%) than in manual-SVF (39.96 ± 6.37%), suggesting a high ASC yield in the CCD system. The EC content was higher in manual-SVF (21.00 ± 2.55%) than in CCD-SVF (11.61 ± 7.24%), whereas the BC contents were similar (32.17 ± 6.16% and 32.87 ± 8.57%, respectively) (Fig. 3).

Adipose tissue contains a variety of cells, including mature adipocytes, ASCs, vascular ECs, pericytes, fibroblasts, and tissue resident macrophages (Yoshimura et al. 2006). One gram of adipose tissue contains about 1 million adipocytes, 1 million ASCs, 1 million vascular ECs, and 1 million other cells (Suga et al. 2008; Eto et al. 2009). The nucleated cell yield and cell components of SVF isolated from lipoaspirate vary by patient and donor site (Tsekouras et al. 2017; Caspar-Bauguil et al. 2005; Di Taranto et al. 2015; Varghese et al. 2017) and isolation methods (Aronowitz and Ellenhorn 2013). Stable isolation of SVF as a viable and ASC-rich fraction is difficult, and thus, the efficiency of SVF cell isolation may be an important advance.

Our results show that SVF obtained by the CCD system is comparable to that obtained by the manual method. In sterility tests, no bacteria were detected in CCD-SVF (Table 2). In addition to its ability to remove collagenase, the sterility of CCD-SVF indicates the safety of this system. The CCD system in this study was standardized and processing steps were simplified by semi-automating cell processing, and the risk of contamination was reduced by using a closed circuit. Cells for clinical use must be processed using standardized procedures under strict clean management based on the “Pharmaceutical and Medical Device Act” and the “Act on Securing Safety of Regenerative Medicine” in Japan. An automated cell isolation system reduces the risk of human errors, training for technicians, variations in technician skills, and the effort and cost of managing aseptic facilities. The CCD system in this study is not a fully integrated SVF processing device, but its potential as a semi-automatic device is evident, and further improvement and development of this system are likely.

**Table 2:** The sterility in the SVF processed by CCD system

Donor ID	NCs (×10^5^ cells/mL Fat)	ASCs contents (%)	viability (%)	bacteria
8	4.3	46.0	83.6	negative
9	5.9	56.2	79.8	negative
10	5.9	45.3	74.7	negative

Abbreviations: NCs, nucleated cells. ASCs, adipose-derived stromal stem cells (CD31-/CD34+/CD45- cells)

**Fig. 4:**
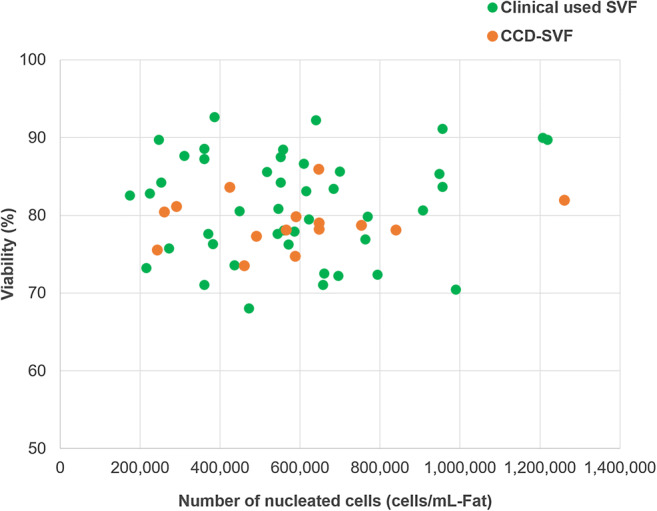
Number of nucleated cells and viability of SVFs used in CAL treatment clinically (green circle) and processed by the CCD system (orange circle)

We compared SVFs processed by the CCD system (*n* = 14) with SVFs used in CAL treatment at the Cellport Clinic Yokohama (January to December 2018, *n* = 44). The CCD-SVFs processed in this study were at the equivalent level to clinical SVFs in terms of the number of nucleated cells and cell viability (Fig. 4). The number of nucleated cells isolated from fat by the manual method varies among donors, and the number of nucleated cells isolated from fat by CCD was within the range of variation. CCD-SVF was similar to SVF used in the clinic, and had comparable properties to those obtained in automated and manual processing in previous reports (Table 3). These results suggest that SVF isolation using the CCD system is appropriate.

**Table 3:** Studies on SVF isolation from adipose tissue

	Processing type	Number of samples	NCs (×10^5^cells)	Range of NCs (×10^5^cells)	Viability (%)	Range of viability
**CCD system**	**Semi-automated**	**n = 14**	**5.81 ± 2.57** (/mL Fat)	**2.42–12.61**	**79.1 ± 0.0**	**73.5–85.9**
Hicok and Hedrick (2011)	manual	*n* = 266	2.3 ± 1.2 (/mL Fat)	0.2–6.8	N/A	N/A
Fraser et al. (2014)	automated	*n* = 31	3.6 ± 1.8(/g Fat)	1.5–7.7	84.7	71.3–95.3
Kamakura and Ito (2011)	automated	*n* = 20	3.42 ± 1.39 (/g Fat) ^⁕^	N/A	85.3 ± 6.2	N/A
Lin et al. (2008)	automated	*n* = 6	2.95 ± 0.99 (/mL Fat)	1.89–4.50	86.6 ± 3.5	80.6–90.0
Doi et al. (2013)	automated	n = 3	7.02 ± 1.89 (/ml Fat)	N/A	80.7 ± 7.1	N/A
	manual	n = 3	7.01 ± 2.43(/ml Fat)	N/A	82.4 ± 7.7	N/A
Aust et al. (2004)	manual	*n* = 18	0.40 ± 0.21 (/mL Fat) ^⁕^	N/A	93.9 ± 3.3	N/A
Aronowitz and Ellenhorn (2013)	automated	*n* = 5	2.41 (/g Fat) ^⁕^	N/A	N/A	N/A
Mitchell et al. (2006)	manual	n = 14	3.09 ± 1.40 (mL Fat)	N/A	N/A	N/A

Abbreviations: NCs, nucleated cells. N/A, not available. ^⁕^viable nucleated cells

## Conclusions

The CCD system is a new SVF isolation method that uses a closed cell washing concentration device with a hollow fiber module. It is as reliable as manual isolation and may also be useful for CAL, and is likely to be of help in development of regenerative medicine.

## Supplementary Information

ESM 1 Supplemental Figure. Cell morphology after washing. Jurkat cell that is stable cell line processed by CCD (left) and by centrifugation (right). Aggregation of cells (yellow circle) was observed in the centrifugation procedure. (PNG 1132 kb)

High Resolution (TIF 15553 kb)

## Data Availability

All data are included in the manuscript.
