# Supplementary figures and images for: A novel method for processing adipose-derived stromal stem cells using a closed cell washing concentration device with a hollow fiber membrane module
Source: Biomed Microdevices. 2021 Jan 6;23(1):3. doi: 10.1007/s10544-020-00541-0 (PMC7788025; doi:10.1007/s10544-020-00541-0)

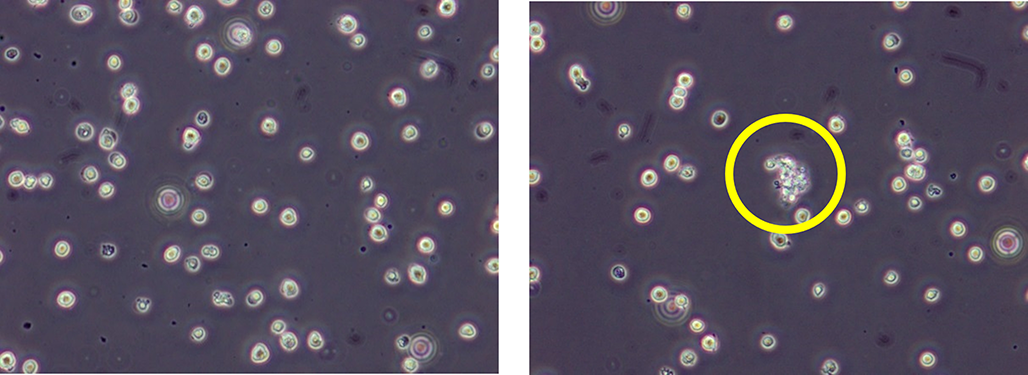

Supplement: Supplementary file 1 — Supplemental Figure. Cell morphology after washing. Jurkat cell that is stable cell line processed by CCD (left) and by centrifugation (right). Aggregation of cells (yellow circle) was observed in the centrifugation procedure. (PNG 1132 kb) [file 10544_2020_541_Fig5_ESM.png]

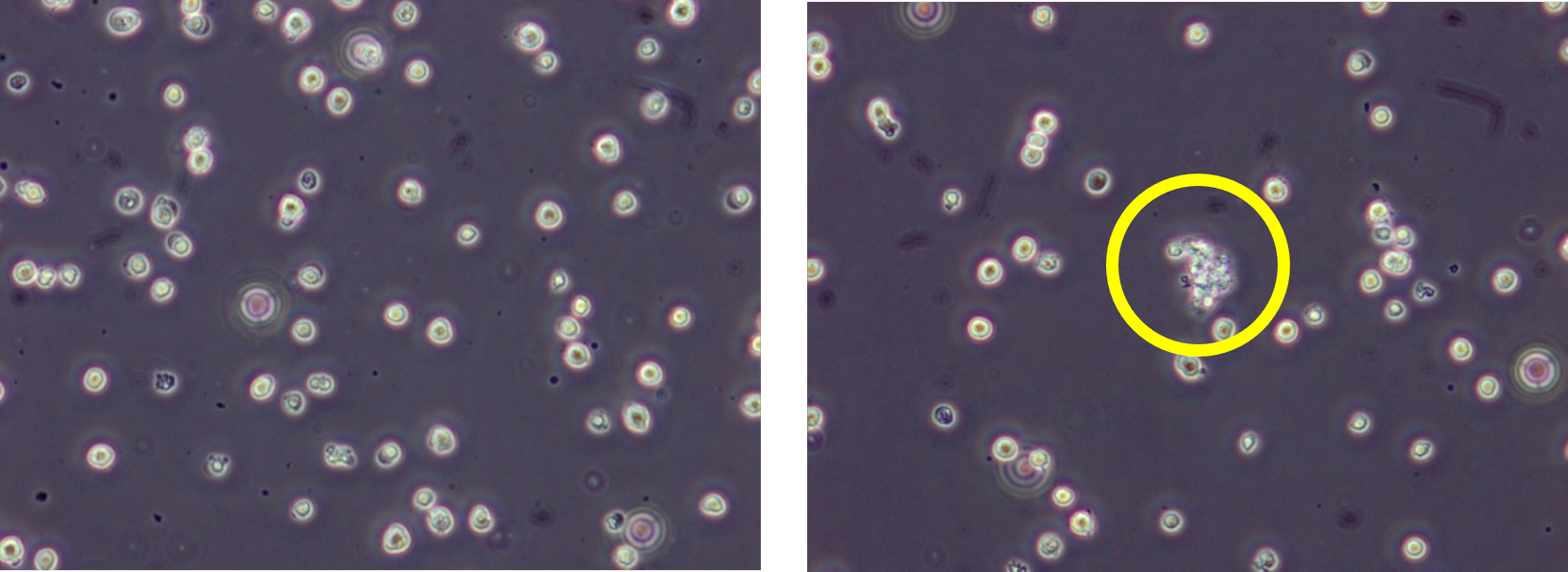

Supplement: Supplementary file 2 — High Resolution (TIF 15553 kb) [file 10544_2020_541_MOESM1_ESM.tif]
